# Supplementary material for: Splice-Junction-Based Mapping of Alternative Isoforms in the Human Proteome
Source: Cell Rep. Author manuscript; Available in PMC 2020 Jan 15. (PMC6961840; doi:10.1016/j.celrep.2019.11.026)
Supplement: 2 [file NIHMS1546469-supplement-2.zip › DF1/PXD010154/Testis/Testis_8_UQCRH_MLTESGDPEEDPLTTVR.pdf]

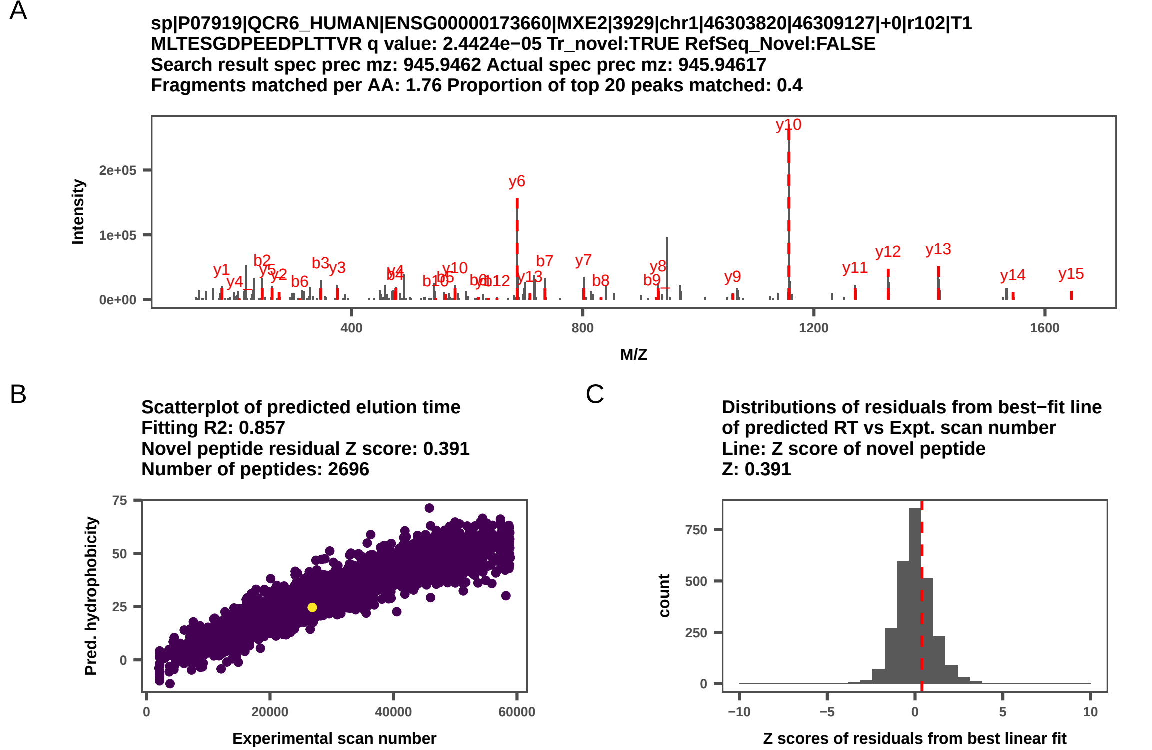
A
sp|P07919|QCR6_HUMAN|ENSG00000173660|MXE2|3929|chr1|46303820|46309127|+0|r102|T1
MLTESGDPEEDPLTTVR q value: 2.4424e−05 Tr_novel:TRUE RefSeq_Novel:FALSE
Search result spec prec mz: 945.9462 Actual spec prec mz: 945.94617
Fragments matched per AA: 1.76 Proportion of top 20 peaks matched: 0.4
Intensity
2e+05
1e+05
0e+00
y10
y6
y1
y4
b2
y5
y2
b6
b3
y3
b4
y4
b10
b5
y10
y12
y13
b7
y7
b8
y8
b9
y9
y11
y12
y13
y14
y15
400
800
1200
1600
M/Z
B
Scatterplot of predicted elution time
Fitting R2: 0.857
Novel peptide residual Z score: 0.391
Number of peptides: 2696
Pred. hydrophobicity
75
50
25
0
0
20000
40000
60000
Experimental scan number
C
Distributions of residuals from best−fit line
of predicted RT vs Expt. scan number
Line: Z score of novel peptide
Z: 0.391
count
750
500
250
0
−10
−5
0
5
10
Z scores of residuals from best linear fit
